# Imaging Biomarkers for the Diagnosis and Prognosis of Neurodegenerative Diseases. The Example of Amyotrophic Lateral Sclerosis

**DOI:** 10.3389/fnins.2018.00784

**Published:** 2018-10-25

**Authors:** Miguel Mazón, Juan Francisco Vázquez Costa, Amadeo Ten-Esteve, Luis Martí-Bonmatí

**Affiliations:** ^1^Radiology and Biomedical Imaging Research Group (GIBI230), La Fe University and Polytechnic Hospital and La Fe Health Research Institute, Valencia, Spain; ^2^Neuromuscular Research Unit, Instituto de Investigación Sanitaria la Fe (IIS La Fe), Valencia, Spain; ^3^ALS Unit, Department of Neurology, Hospital Universitario y Politécnico La Fe, Valencia, Spain; ^4^Centro de Investigación Biomédica en Red de Enfermedades Raras (CIBERER), Valencia, Spain

**Keywords:** amyotrophic lateral sclerosis, imaging biomarkers, neurodegenerative disorders, brain MR image analysis, a structured report

## Abstract

The term amyotrophic lateral sclerosis (ALS) comprises a heterogeneous group of fatal neurodegenerative disorders of largely unknown etiology characterized by the upper motor neurons (UMN) and/or lower motor neurons (LMN) degeneration. The development of brain imaging biomarkers is essential to advance in the diagnosis, stratification and monitoring of ALS, both in the clinical practice and clinical trials. In this review, the characteristics of an optimal imaging biomarker and common pitfalls in biomarkers evaluation will be discussed. Moreover, the development and application of the most promising brain magnetic resonance (MR) imaging biomarkers will be reviewed. Finally, the integration of both qualitative and quantitative multimodal brain MR biomarkers in a structured report will be proposed as a support tool for ALS diagnosis and stratification.

## Introduction

Amyotrophic lateral sclerosis (ALS), also known as motor neuron disease, comprises a heterogeneous group of fatal neurodegenerative disorders of largely unknown etiology characterized by the degeneration of upper motor neurons (UMN) and/or lower motor neurons (LMN) in the primary motor cortex, brain stem, and spinal cord (Kiernan et al., 2011; Al-Chalabi et al., 2016). Classically, ALS has been considered a neurodegenerative disease that exclusively affects motor neurons, leading to progressive muscle weakness. However, convincing evidence supports the notion that it is a multisystem disease not limited to motor areas alone but involving extramotor areas as well (Al-Chalabi et al., 2016) and causing a variable degree of cognitive, behavioral or dysautonomic symptoms (Al-Chalabi et al., 2016; Arlandis et al., 2017).

The median age of onset is 55 years and the incidence and prevalence of ALS are 1–2 and 4–6 per 100,000 each year, respectively, with a lifetime ALS risk of 1/600–1/1,000 (Beghi et al., 2006).

ALS is sporadic in 90% of cases and show familial inheritance in the remaining 10% (Al-Chalabi et al., 2016), although some prospective studies in which genealogies have been actively investigated, the prevalence of a family history increases to 17–23% (Andersen and Al-Chalabi, 2011). The causative mutation is known in approximately 60% of familial cases. In European populations, the most common mutation is the C9ORF72 expansion followed by mutations in the SOD1, TARDBP, and FUS genes (Zou et al., 2017). The cause of sporadic ALS remains in most cases unknown, although mutations in familial ALS genes have also been reported (Zou et al., 2017).

There are multifactorial pathogenic processes underlying ALS which are not yet fully determined. Cellular events such as oxidative stress, mitochondrial dysfunction, excitotoxicity, protein aggregation, impaired axonal transport, neuroinflammation, and dysregulated RNA signaling, contribute to the pathobiology of ALS (Ling et al., 2013).

The hallmark histopathological features of ALS are motor neuron loss in the motor cortex and spinal cord, and corticospinal tract degeneration (Ferraiuolo et al., 2011). Gross pathological changes in the nervous system include (Ferraiuolo et al., 2011): (1) atrophy of the precentral gyrus; (2) shrinkage, sclerosis and pallor of the corticospinal tracts; and (3) thinning of the spinal ventral roots and hypoglossal nerves. Microscopic changes include (Ferraiuolo et al., 2011; Kwan et al., 2012): (1) depletion of the spinal cord motor neurons and giant pyramidal neurons in the motor cortex; (2) diffuse astrocytic gliosis of spinal gray matter; (3) presence in surviving lower motor neurons of ubiquitinated inclusion bodies; (4) atrophic and basophilic changes in surviving motor neurons; (5) variable astrocytic gliosis in the gray matter of the motor cortex and underlying subcortical white matter; (6) cytoplasmic aggregate inclusions within glial cells; (7) evidence of microglial activation in pathologically affected areas with iron accumulation as ferritin sequestered in activated microglia in the motor cortex in the middle and deep layers, sparing the superficial layers.

ALS symptoms usually begin in the distal limb or bulbar musculature, and typically spread to contiguous body regions (Ravits and La Spada, 2009), causing progressive muscle weakness and eventually leading to death by respiratory insufficiency. The median survival from symptom onset is 2–3 years, but there are great individual variations (Kiernan et al., 2011). The phenotypic expression and prognosis of ALS is, therefore, highly heterogeneous and mainly determined by five elements (Ravits and La Spada, 2009; Al-Chalabi et al., 2016): (1) body region of onset (bulbar vs. spinal); (2) relative mix of UMN and LMN involvement; (3) progression rate; (4) age of onset, and (5) the presence of extra-motor symptoms. The dominance of UMN vs. LMN signs is variable, with extremes of UMN involvement termed primary lateral sclerosis (PLS) and those of LMN involvement, termed progressive muscular atrophy (PMA) (Al-Chalabi et al., 2016).

The diagnosis of ALS remains clinical (Al-Chalabi et al., 2016), based on the detection of clinical UMN signs, clinical and electrophysiological LMN signs and the exclusion of ALS mimics (Brooks et al., 2000; de Carvalho et al., 2008). Current diagnostic criteria (the revised El Escorial criteria or the more recent Awaji criteria) classify patients in three certainty levels (definite, probable, possible) according to the number of regions (bulbar, cervical, dorsal and lumbar) with UMN and LMN impairment (Brooks et al., 2000; de Carvalho et al., 2008). These criteria are intended to their use in research and clinical trials, not in the clinical practice, and consequently, show a high specificity but a very limited sensitivity (Al-Chalabi et al., 2016).

The phenotypic heterogeneity of ALS, together with the absence of a reliable UMN biomarker results in a long diagnostic delay (of about 12 months or even higher in incomplete phenotypes) (Zoccolella et al., 2006; Vázquez-Costa et al., 2018a), which limits early initiation of potential neuroprotective treatments. Moreover, they difficult both the recruitment and the results interpretation of clinical trials (Agosta et al., 2010a). Consequently, there is an urgent need of biomarkers (especially UMN ones) in ALS for both clinical practice and research.

The ideal UMN biomarkers should shorten the diagnostic delay in ALS while decreasing the number of misdiagnosed cases. They should improve our understanding of ALS pathophysiology and phenotypes, and the mechanisms underlying the progressive degenerative process. Finally, they should be able to monitor the disease progression for both clinical, as marker of patients’ prognosis, and research settings, as biological markers for assessing the efficacy of experimental treatments (Chiò et al., 2014).

## Imaging Biomarkers: General Concepts

In magnetic resonance (MR), the radiological diagnosis is usually based on the qualitative integration and assessment of those imaging findings obtained from tailored examinations performed in specific clinical situations together with detailed protocols trying to highlight those expected aspects related to the disease. The digital nature of the obtained MR signal and image reconstructions allows to calculate voxelwise derived quantitative data related to the picture features attributes, such as texture or morphological descriptors, and biological model parameters, such as gray matter iron deposits.

The implementation of quantitative imaging biomarkers in clinical practice needs both a definition of the radiomic pipeline and the use of structured reports. The process involves study acquisition, image preparation, volume of interest definition, data extraction, data analysis, and outcomes presentation.

Computational medical imaging tools are used to define the volumes of interest and the relevant data to be extracted. This radiological science studies the properties and behavior of the tissues by analyzing the digital acquired images in an attempt to describe those imaging facts which are relevant to the patient. The extracted data should be accurate and truthful. This extracted radiomic high-dimensional pieces of information are in a mineable form, allowing to build descriptive, predictive and prognostic models from the medical image features and their related parameters (Martí Bonmatí et al., 2012).

If the calculated parameters are objectively measured and behave as indicators of normal biological processes, pathological changes, or pharmaceutical responses, they are recognized as biomarkers. Imaging biomarkers will be, therefore, those subrogated features and parameters extracted from medical images that give quantitative information as the regional distribution and magnitude of the depicted characteristic. As the process is voxelwise, the calculated computational images are resolved in space (as a parametric analysis) and time (as a longitudinal evaluation).

The main objects of study are: the predisposition and risk of suffering a disease; the screening and early diagnosis in predisposed subjects before symptoms appear; precise diagnosis and differential diagnosis of the suspected disease; individualized lesion grading; disease classification and staging; lesion phenotyping; patient prognosis and expected natural history; selection of best treatment options; evaluation of possible image guided intervention; treatment effects evaluation and response criteria; follow-up and patients’ surveillance after treatment.

The main clinical endpoints to check for the appropriateness of the defined biomarkers are related to patients’ status (survival, quality of life, symptoms relive) and disease situation (lesion phenotype, progression).

In opposition to multimodality imaging, where two modalities are combined to compensate for the disadvantages of each one, while taking advantage of their individual strengths such as in PET/CT, the multiparametric imaging approach is the result of a multidimensional data reduction techniques applied to those relevant radiomic parameters, on a voxel-by-voxel basis, to compensate for the disadvantages of single parameter analysis.

Before implementing in the clinical practice, variability has to be settled. Repeatability, defined as the variability evaluation within patients (test-retest data sets), is the result of acquisition errors and bio/physiological changes, while reproducibility can be considered as the variability within the methods or procedures (independent data sets), evaluating the influence of the different MR acquisition techniques, vendor scanners and hospital centers. The expected precision values (±) are an indication of these repeatability and reproducibility uncertainties.

An important source of reproducibility inaccuracies is related to the fact that the image reconstructed signal coming from the voxel is a mixture of complex signals from different structures, components and properties. Therefore, for an image derived measurement to be representative of a physical reality, it must have an unambiguous and truly relation with the process it measures. The control of biases, both at the acquisition and postprocessing level is critical.

The precision of the measurements is influenced by the image acquisition parameters (different centers, vendors, protocols, patient preparation) and image processing methods (computer algorithm, influence of human interaction). On the other hand, biomarker’s accuracy is defined by phantom calibration (relative error to synthesized ground truth) and pathological truth (relative error to current gold standards, such as biopsy). The clinical endpoints can be evaluated in a short term (lesion and disease diagnostic and therapeutic values) or long patient’s prognostic value. Validated imaging biomarkers should at least have been validated in one precision, accuracy and clinical background before implementation.

The use or artificial intelligence, with machine learning solutions (including deep learning, random forests, support vector machines and neural networks), will surely affect this process by allowing automation in the segmentation process, detection of tissue changes and patterns, and characterization of patients. Machine learning may be used to build, train and validate models to aid in prediction and early stratification of patients from their macroscopic based MR imaging features. These methods are not exclusive for MR imaging, and they can be used with other imaging techniques (Segovia et al., 2017a,b; Ortiz et al., 2019) and even with clinical, molecular, and genetic biomarkers applied to build a model of the pathology (Latourelle et al., 2017). Some examples on this approach highlighting the benefits of this computer aided diagnosis (CAD) systems analysis is beyond the scope of this review but it’s worth mentioning some works as the following (Sengur et al., 2017; van der Burgh et al., 2017; Martinez-Murcia et al., 2018; Yamashita et al., 2018).

## Conventional Brain MR Imaging and Qualitative MR Imaging Biomarkers in ALS

Conventional MR imaging of the brain and, occasionally, the spinal cord is mainly performed to rule out other pathologies that may also present with UMN and/or LMN signs. Potential ALS mimickers include cerebral lesions, skull base tumors, cervical spondylotic myelopathy, conus abnormalities, and thoraco/lumbo/sacral radiculopathy (Agosta et al., 2010a). The revised criteria of the World Federation of Neurology Research Group on Motor Neuron Diseases recommend conventional imaging in “clinically probable” or “possible ALS,” while imaging is not consider required in cases of “clinically definite” disease with a bulbar or a pseudobulbar onset (Brooks et al., 2000; de Carvalho et al., 2008; Agosta et al., 2010a).

**FIGURE 1:**
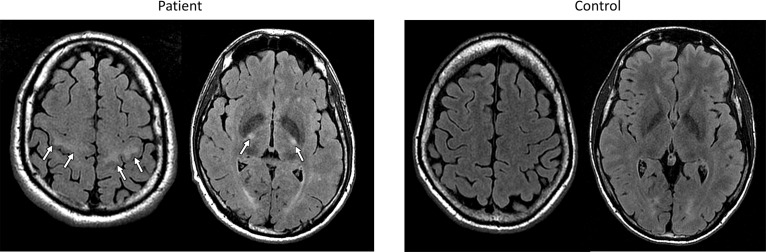
FLAIR MR images from an exemplary patient with ELA (left) and a control subject (right) evaluated in our center, showing bilateral hyperintensities of the corticospinal tract in subcortical white matter and posterior limb of capsula internal in the ELA patient.

Several studies have reported abnormal findings on conventional MR images in patients with ALS. Standard turbo spin echo T2-weighted and FLAIR sequences may reveal hyperintensity of the corticospinal tract (CST) from the centrum semiovale to the brain stem (Agosta et al., 2010a; Foerster et al., 2013b; Figure 1). These CST hyperintensities might reflect areas of reduced axonal and myelin density (Yagishita et al., 1994) and have been associated with UMN signs and bulbar onset (Vázquez-Costa et al., 2018b). However, the frequency of CST lesions ranges widely across studies, from 15 to 95% (Hecht et al., 2001; Huynh et al., 2016; Jin et al., 2016), depending on the used sequences (FLAIR, T2, proton density) and the analyzed region of the CST. More remarkably, CST hyperintensities have also been described in other conditions and even in healthy subjects (Mirowitz et al., 1989; Agosta et al., 2010a; Huynh et al., 2016).

A low signal intensity rim in the precentral cortex on the T2-weighted images can also be found in ALS patients (Kwan et al., 2012; Vázquez-Costa et al., 2018b). This is probably related to iron deposition, since increased iron deposition, in the form of ferritin in activated microglia in the middle and deep layers of the motor cortex, has been proved in histologic analysis and postmortem examinations (Oba et al., 1993; Kwan et al., 2012). Moreover, in ALS, many lines of evidence indicate that there is an abnormal iron homeostasis inducing excessive oxidative stress in the motor neurons (Jomova et al., 2010; Petri et al., 2012).

MR techniques with specific susceptibility such as T2, T2^∗^, relaxometry, magnetic field correlation imaging and susceptibility-weighted imaging (SWI) are especially sensitive to iron detection.

Iron causes a concentration-dependent irregularity in the local magnetic field and a signal darkening on T2 and T2^∗^-weighted images that is greater with increasing magnetic field strength. Initial studies noted a shorter T2 time in the motor cortex in a subset of ALS patients (Ishikawa et al., 1993; Oba et al., 1993; Cheung et al., 1995; Waragai, 1997; Anderson et al., 2001; Hecht et al., 2002). However, such change was neither sensitive nor specific. T2^∗^ weighted gradient-echo sequences have greater sensitivity to detect the presence of iron in different tissues (Thorpe et al., 1996; Hecht et al., 2005). Several studies have shown that the T2^∗^ shortening corresponds histologically to iron deposition in the microglia of the motor cortex (Oba et al., 1993; Kwan et al., 2012). Furthermore, other T2^∗^ studies have developed a semi-quantitative assessment of these signal changes, showing good diagnostic accuracy and a correlation between the degree of the signal hypointensity and the severity of motor impairment and disease progression rate, specially with high-resolution MR imaging at ultra-high field strength (Kwan et al., 2012; Ignjatović et al., 2013; Cervo et al., 2015). Finally, a single follow-up study has highlighted the progression of a T2 hypointense area in the motor cortex after 6 months, which was strongly and negatively correlated with the severity of the disease (Ignjatović et al., 2013).

SWI is a three-dimensional gradient echo sequence with full flow compensation and high resolution that enhances image contrast by using the susceptibility differences between tissues, which aids in the identification of paramagnetic non-heme iron. SWI is created by combining both magnitude and phase in the gradient echo data, iron appears hypointense and it can be easily assessed. The main advantage of SWI as opposed to T2^∗^-weighted images is to provide a higher contrast image, more sensitive to iron overload. Consequently, SWI has been proved superior to T2 and T2^∗^-weighted images to detect the histologically proven iron deposition in the precentral cortex in ALS (Adachi et al., 2015). The qualitative evaluation of iron-related changes on SWI has obtained poor diagnostic accuracy (Sheelakumari et al., 2016). Conversely, in our experience, a careful semi-quantitative assessment of the SW images in a 3T MR shows good sensitivity and very good specificity for ALS diagnosis. Moreover, it may lead to improve the sensitivity of the research diagnostic criteria and could help to the diagnosis in some ALS phenotypes in the clinical. A recent study has used the power of the reconstruction with phase difference enhanced imaging in a 3D flow-compensated multi-echo spoiled gradient echo sequence to assess the motor cortex; this imaging method found a very high diagnostic accuracy (0.94) (Kakeda et al., 2016).

**FIGURE 2:**
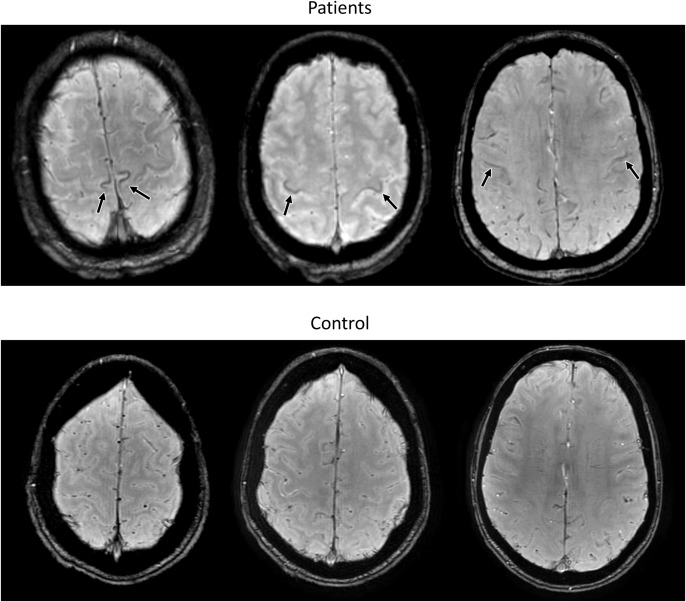
Susceptibility Weighted MR images from 3 different exemplary ELA patients (upper row) and a control subject (lower row) for comparison, evaluated in our center. Note the iron-related hypointensities in the motor cortex at the lower limbs, upper limbs, and bulbar region (from left to right) in the patients with ELA.

The motor cortex can be subdivided into three subregions, which correspond to the cortical representation of lower limbs, upper limbs and bulbar musculature, in each hemisphere. Following this semi-quantitative methodology, iron-related hypointensities were found to be a reliable marker of UMN degeneration and were more frequent in bulbar onset patients, independently of the mutation status (Vázquez-Costa et al., 2018b; Figure 2). Moreover, their intensity and extent in the different motor homunculus regions (lower limbs, upper limbs, and bulbar) were linked to the symptoms onset site, suggesting that the regional measurement of iron-related hypointensities following the motor homunculus could be used as a measure of disease progression (Vázquez-Costa et al., 2018b).

To sum up, the reported sensitivity and specificity of the qualitative MR biomarkers for the ALS diagnosis varies widely among studies, probably due to methodological differences. However, the semi-quantitative assessment of the CST on FLAIR and especially of the motor cortex on SWI seems to be a quick imaging tool that can help to differentiate ALS patients from healthy individuals. In the MR evaluation of an ALS patient, we suggest an assessment of the CSTs in the subcortical precentral white matter and in the posterior limb of the internal capsules on FLAIR images; and an assessment of the motor cortex subregions corresponding to the bulbar, upper limbs and lower limbs motor neurons on SWI. For each subregion, scores from 0 to 2 are assigned, based on its signal intensity (Vázquez-Costa et al., 2018b). Following this semi-quantitative evaluation, a global score for the CST and another for the motor cortex intensity are provided in the structured report.

## Quantitative Brain MR Imaging Biomarkers in ALS

Given the abovementioned limitations of the qualitative MR biomarkers, an objective, sensitive and quantitated imaging marker of UMN dysfunction is needed, with relevance for both patohophysiologic investigation and clinical practice (Agosta et al., 2018). Brain MR imaging is a widely available non-invasive technique, able to provide radiomic data that might be appropriate candidates for UMN biomarkers. In the last years, many different MR techniques have been used with varying success. They include structural imaging (voxel-based morphometry and cortical thickness), diffusion tensor imaging (DTI, anisotropy), spectroscopy (MRS), iron-sensitive sequences (T2^∗^ and SWI) and functional MR imaging (fMRI) (Table 1 sumarizes the results of the most significant studies).

**Table 1:** Main results of most significant studies.

**Conventional brain MR imaging and qualitative MR imaging biomarkers**	
Hyperintensity of the corticospinal tract on FLAIR/T2	Hecht et al., 2001; Agosta et al., 2010a; Foerster et al., 2013b; Huynh et al., 2016; Jin et al., 2016
Hypontensity of the motor cortex on SWI	Adachi et al., 2015; Sheelakumari et al., 2016; Vázquez-Costa et al., 2018b
**Structural magnetic resonance imaging**	
Atrophy in the precentral gyri (VBM)	Ellis et al., 2001; Chang et al., 2005; Kassubek et al., 2005; Grosskreutz et al., 2006; Agosta et al., 2007; Mezzapesa et al., 2007; Thivard et al., 2007; Menke et al., 2014; Zhang et al., 2014; Devine et al., 2015
No atrophy in the precentral gyri (VBM)	Kiernan and Hudson, 1994; Ellis et al., 2001; Mezzapesa et al., 2007; Raaphorst et al., 2015
Atrophy in extra-motor regions (VBM)	Chang et al., 2005; Kassubek et al., 2005; Agosta et al., 2007, 2012; Mezzapesa et al., 2007; Thivard et al., 2007; Bede et al., 2013; Abdulla et al., 2014; Zhang et al., 2014; Christidi et al., 2018
Cortical thinning of the primary motor cortex (SBM)	Roccatagliata et al., 2009; Verstraete et al., 2010, 2012; Agosta et al., 2012; Walhout et al., 2015; Al-Chalabi et al., 2016
Cortical thinning of extra-motor regions (SBM)	Verstraete et al., 2012; Mioshi et al., 2013; d’Ambrosio et al., 2014; Schuster et al., 2014a; Walhout et al., 2015
**Diffusion tensor imaging**	
Decreased FA and increased ADC of the corticospinal tract	Ellis et al., 1999; Sage et al., 2007, 2009; Schimrigk et al., 2007; Iwata et al., 2008, 2011; Yin et al., 2008; Ciccarelli et al., 2009; Nickerson et al., 2009; Filippini et al., 2010; Stagg et al., 2013; Foerster et al., 2014; Sarica et al., 2014; Rosskopf et al., 2015; Tang et al., 2015
Decreased FA in the corpus callosum	Sach et al., 2004; Agosta et al., 2007; Sage et al., 2007, 2009; Ciccarelli et al., 2009; Senda et al., 2009; Filippini et al., 2010; Zhang et al., 2011; Rose et al., 2012; Shen et al., 2016
Decreased FA in extra-motor regions	Abe et al., 2004; Sach et al., 2004; Agosta et al., 2007; Sage et al., 2007, 2009; Thivard et al., 2007; Ciccarelli et al., 2009; Filippini et al., 2010; Keil et al., 2012; Prell et al., 2013; Rosskopf et al., 2015
**Magnetic resonance spectroscopy**	
Decrease in absolute NAA concentrations in the motor cortex.	Gredal et al., 1997; Rooney et al., 1998; Bradley et al., 1999; Pohl et al., 2001b; Sarchielli et al., 2001; Schuff et al., 2001; Block et al., 2002; Suhy et al., 2002; Kalra et al., 2006a,c; Mitsumoto et al., 2007; Han and Ma, 2010; Cervo et al., 2015; Liu et al., 2015
Decrease NAA/Cr in the motor cortex.	Pioro et al., 1994; Jones et al., 1995; Block et al., 1998, 2002; Rooney et al., 1998; Abe et al., 2001; Rule et al., 2004; Yin et al., 2004; Kalra et al., 2006a,b; Mitsumoto et al., 2007; Charil et al., 2009; Lombardo et al., 2009; Han and Ma, 2010; Pyra et al., 2010; Sivák et al., 2010
Decrease in NAA/Cho in the motor cortex.	Pioro et al., 1994; Jones et al., 1995; Giroud et al., 1996; Block et al., 1998, 2002; Rooney et al., 1998; Pohl et al., 2001a; Suhy et al., 2002; Rule et al., 2004; Kalra et al., 2006a; Pyra et al., 2010
Decrease in NAA/(Cr+Cho) in the motor cortex.	Rooney et al., 1998; Suhy et al., 2002; Rule et al., 2004
Other altered metabolites in the motor cortex.	Block et al., 1998; Bowen et al., 2000; Kalra et al., 2006a; Lombardo et al., 2009; Han and Ma, 2010; Foerster et al., 2012a, 2014
Altered metabolites in the CST and extra-motor regions	Rooney et al., 1998; Bowen et al., 2000; Abe et al., 2001; Schuff et al., 2001; Rule et al., 2004; Yin et al., 2004; Han and Ma, 2010; Pyra et al., 2010; Sivák et al., 2010; Sharma et al., 2011; Sudharshan et al., 2011; Govind et al., 2012; Foerster et al., 2014
**Quantitative iron imaging**	
Iron deposition in motor cortex	Costagli et al., 2016; Donatelli et al., 2018a
Iron deposition in CST and extra-motor regions	Minnerop et al., 2009; Langkammer et al., 2010a; Prell et al., 2015
**Functional magnetic resonance (fMR) imaging**	
Cerebral activation alterations during motor tasks (task-based fMRI)	Konrad et al., 2002, 2006; Schoenfeld et al., 2005; Han and Ma, 2006; Lulé et al., 2007b; Stanton et al., 2007; Kollewe et al., 2011; Mohammadi et al., 2011, 2015; Cosottini et al., 2012
Cerebral activation alterations with other tasks (task-based fMRI)	Abrahams et al., 2004; Lulé et al., 2007a, 2010; Palmieri et al., 2010; Passamonti et al., 2013
Decreased functional connectivity within the sensorimotor network (Resting State fMRI)	Mohammadi et al., 2009a; Jelsone-Swain et al., 2010; Tedeschi et al., 2012; Fekete et al., 2013; Zhou et al., 2013
Abnormalities in other networks (Resting State fMRI)	Mohammadi et al., 2009a; Jelsone-Swain et al., 2010; Luo et al., 2012; Zhou et al., 2013
Increased network coherence in the somatosensory and extra-motor areas (Resting State fMRI)	Agosta et al., 2011, 2013, 2014; Douaud et al., 2011; Luo et al., 2012; Tedeschi et al., 2012; Fekete et al., 2013; Zhou et al., 2013


### Structural Magnetic Resonance Imaging

Brain morphological structure can be examined using high spatial resolution MR imaging techniques. Automated analysis techniques allow to segment and quantify gray and white matter volume and morphology using T1-weighted images. The two main morphometric analysis methods are voxel-based morphometry (VBM) and surface-based morphometry (SBM) (Verstraete and Foerster, 2015). These whole-brain techniques do not require an a priori assumption of the brain districts affected by disease and are able to determine the structural anomalies directly from the data in an unbiased way (Mezzapesa et al., 2007).

VBM is an objective approach that enables a voxel-wise estimation of the local amount of a specific brain component (gray or white matter) (Kurth et al., 2015). VBM compares the classification of each voxel in patients and controls, resulting in a regional assessment of gray or white matter “density” (not strictly equivalent to atrophy in the histological sense) (Ashburner and Friston, 2000).

VBM studies have yielded inconsistent results regarding the presence of abnormalities in the primary motor cortex, including the premotor cortex, and the extent of non-motor cortex atrophy in ALS patients (Chiò et al., 2014). Although most studies have evidenced atrophy changes in the precentral gyri (Ellis et al., 2001; Chang et al., 2005; Kassubek et al., 2005; Grosskreutz et al., 2006; Agosta et al., 2007; Mezzapesa et al., 2007; Thivard et al., 2007; Menke et al., 2014; Zhang et al., 2014; Devine et al., 2015), others have failed (Kiernan and Hudson, 1994; Ellis et al., 2001; Mezzapesa et al., 2007; Raaphorst et al., 2015). Moreover, the distribution of the involvement of the extra-motor cortex varies considerably among the different studies. The involved regions include areas of the temporal cortex, the hippocampus, the parietal cortex and the insula (Chang et al., 2005; Kassubek et al., 2005; Agosta et al., 2007; Mezzapesa et al., 2007; Thivard et al., 2007; Abdulla et al., 2014; Zhang et al., 2014; Christidi et al., 2018). Low volume of occipital gray matter, cerebellum, thalamus and the caudate nucleus, have been identified, but they are less common (Chang et al., 2005; Mezzapesa et al., 2007; Thivard et al., 2007; Agosta et al., 2012; Bede et al., 2013). There are large discrepancies between studies about the extension of changes, which might be due to different biases, including a small sample size, no control group for comparisons, different disease spectrum, unclear patient characterization, data driven design of the studies without a clinical hypothesis, and no proper control of laterality and symmetry of abnormal changes (Bede and Hardiman, 2014). However, a recent voxel-wise meta-analysis of 29 VBM studies, comprising 638 patients with ALS and 622 healthy controls, (Shen et al., 2016) revealed atrophy mainly in the right precentral gyrus, the left Rolandic operculum, the left lenticular nucleus, and the right anterior cingulate and paracingulate gyri, confirming that brain changes in ALS extend beyond the motor system, even in the absence of overt cognitive deficits.

VBM diverging results in the evaluation of the cortical atrophy distribution in ALS patients has promoted other advanced MR imaging approaches. The SBM algorithm also allows the reconstruction of the cortical surface after by defining gray and white matter boundaries, gray matter volume and cerebrospinal fluid. Afterward, the cortex is parceled based and the cortical thickness, curvature maps, surface area and sulcal depth calculated (Fischl and Dale, 2000). Compared to VBM, SBM has a higher reliability and sensitivity to focal atrophy changes in cortical motor regions, being cortical thickness the key metric to detect ALS changes in the motor cortex (Verstraete et al., 2012).

**FIGURE 3:**
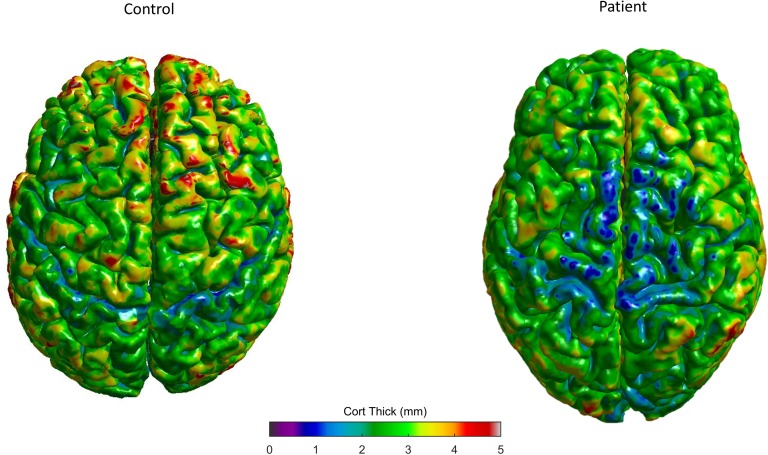
Control subject (left) and exemplary ELA patient (right) 3D cortical thickness surface rendering maps, evaluated in our center, showing the decreased thickness in specific gray matter areas (colors represent different thickness as shown in the bar).

SBM studies have revealed a consistent cortical thinning of the primary motor cortex in ALS (Roccatagliata et al., 2009; Verstraete et al., 2010, 2012; Agosta et al., 2012; Walhout et al., 2015; Al-Chalabi et al., 2016) and PLS patients (Iwata et al., 2011; Kwan et al., 2013), but not in PMA patients (Walhout et al., 2015; Spinelli et al., 2016; Figure 3). Furthermore, bulbar scores and arm functional scores were associated with cortical thickness of corresponding regions of the motor homunculus (Walhout et al., 2015) and a significant correlation was found between thinning within the temporal lobe cortex and a faster disease progression (Verstraete et al., 2012). Extra-motor involvement seems to be related to disease disability, progression, and duration (d’Ambrosio et al., 2014). Moreover, frontotemporal and parietal thinning was more severe in patients with cognitive impairment and ALS than in those with only motor ALS forms (Mioshi et al., 2013; Schuster et al., 2014a). The cortical differences may explain the clinical heterogeneity up to a certain point (Dharmadasa et al., 2018).

Longitudinal MR imaging studies in ALS are challenging, as progressive bulbar and respiratory weakness limit repeated scanning (Turner et al., 2012). Most longitudinal structural MR studies have found a progressive widespread frontotemporal cortical and subcortical atrophy (Agosta et al., 2009; Verstraete et al., 2012; Kwan et al., 2013; Menke et al., 2014, 2018; Schuster et al., 2014b; Walhout et al., 2015; Westeneng et al., 2015). Interestingly, the extra-motor areas seem to have deeper changes with disease progression, probably because the atrophy of the precentral gyrus shows a ceiling effect early in the disease course (Menke et al., 2014; Schuster et al., 2014b; Walhout et al., 2015).

*As a summary*, these findings support morphometric imaging as surrogate marker for assessing UMN impairment. Cortical thickness seems to be the most promising neuroimaging biomarker of ALS. Both morphometric analysis methods, VBM and SBM, are reccomended; and the regional atrophy changes and cortical thickness results should be included in the structured report.

### Diffusion Tensor Imaging

Diffusion-weighted (DW) imaging signal relates to the random Brownian motion of water molecules within an MR voxel. As diffusion is a three-dimensional process, molecular mobility may be limited in some directions, as in white matter anisotropy. This anisotropy effects can be shown with diffusion tensor imaging (DTI) sequences, able to characterize and provide exquisite details on tissue microstructure (Le Bihan et al., 2001). DTI analysis of the fascicular white matter architecture of axons in parallel bundles and their myelin shield reflects the diffusion of water molecules along their main direction. If diffusion gradients are applied in at least 6 non-collinear directions, a diffusion tensor will be calculated for each voxel, being represented by an ellipsoid with its principal axes along the eigenvectors.

Derived main DTI measurements include fractional anisotropy (FA), axial and radial diffusivity, and mean diffusivity. FA is a scalar measurement of the preferred axis of diffusion movement, decreasing as the diffusion of water becomes less restricted to a single axis. FA values are a major indicator of white matter fibers anisotropy, being related to myelin integrity, and density and parallelism of fibers.

Axial diffusivity measures the water diffusion component parallel to axons, thus revealing the gross axonal status, while radial diffusivity assesses water diffusion perpendicular to fibers, being mainly influence by changes in the myelin sheath (Kumar et al., 2013). Mean diffusivity is the average displacement of water molecules within the voxel, ignoring diffusivity non-uniformity by averaging the diffusivity properties from all main axes. Usually, main diffusivity D is also referred to as the apparent diffusion coefficient (ADC).

The computational analysis of DTI metrics can be performed using ROI approaches; whole-brain voxel-wise methods, such as VBM-style analysis or tract-based spatial statistics (Smith et al., 2006); or tractography segmented analyses, which can characterize white matter tracts.

**FIGURE 4:**
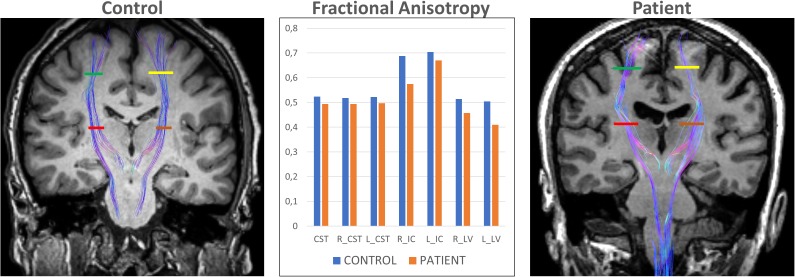
DTI tractography overlaid on coronal T1-weighted images of a control subject (left) and exemplary ELA patient (right) evaluated in our center. The Corticospinal Tract (CST) at 2 different levels allows comparing the differences in Fractional Anisotropy between control subjects and ELA patients. R_CST, right CST; L_CST, left CST; R_IC, right Posterior Limb of Internal Capsule (red line); L_IC, left Posterior Limb of Internal Capsule (brown line); R_LV, right Centrum Semiovale at top of the lateral ventricle (green line); and L_LV, left Centrum Semiovale at top of the lateral ventricle (yellow line).

DTI studies of ALS patients have consistently reported decreased FA and increased ADC of the corticospinal tract (Ellis et al., 1999; Sage et al., 2007, 2009; Schimrigk et al., 2007; Iwata et al., 2008, 2011; Yin et al., 2008; Ciccarelli et al., 2009; Nickerson et al., 2009; Filippini et al., 2010; Stagg et al., 2013; Foerster et al., 2014; Sarica et al., 2014; Rosskopf et al., 2015; Tang et al., 2015; Figure 4). However, patterns of changes along the corticospinal tract differ among studies. The most pronounced decreased FA and increased ADC have been shown in the posterior limb of internal capsule (Ellis et al., 1999; Toosy et al., 2003; Abe et al., 2004; Graham et al., 2004; Cosottini et al., 2005; Sage et al., 2007; Schimrigk et al., 2007; Iwata et al., 2008; Ciccarelli et al., 2009; Senda et al., 2009). Despite the consistency of FA changes found in the CST, its ability to differentiate patients from healthy controls at an individual level, is very poor, as a recent meta-analysis showed (Foerster et al., 2012b).

Several studies have also reported a FA decrease in the corpus callosum (CC) (Sach et al., 2004; Agosta et al., 2007; Sage et al., 2007, 2009; Ciccarelli et al., 2009; Senda et al., 2009; Filippini et al., 2010; Zhang et al., 2011; Rose et al., 2012; Shen et al., 2016), in keeping with historical neuropathological observations (Smith, 1960; Keil et al., 2012). Studies employing a voxel-wise approach were able to investigate differences in FA between ALS patients and controls in regions outside the “classical” motor network and showed reductions in FA in the following extra-motor regions: the frontal lobe (Abe et al., 2004; Sach et al., 2004; Agosta et al., 2007; Sage et al., 2007, 2009; Thivard et al., 2007; Ciccarelli et al., 2009; Filippini et al., 2010; Keil et al., 2012; Rosskopf et al., 2015), the cingulum (Prell et al., 2013), the parietal lobe (Sage et al., 2007; Thivard et al., 2007; Ciccarelli et al., 2009), the temporal lobe (Agosta et al., 2007), parahippocampal areas (Keil et al., 2012), the hippocampus (Sage et al., 2007), the insula (Sage et al., 2007, 2009), the cerebellum (Keil et al., 2012) and the thalamus (Sach et al., 2004; Thivard et al., 2007; Sarica et al., 2014).

Many studies have revealed a relationship with clinical measures. A decreased FA in the corticospinal tracts has been found to be correlated with disease severity (Ellis et al., 1999; Graham et al., 2004; Cosottini et al., 2005; Wang et al., 2006a; Sage et al., 2007, 2009), rate of disease progression (Agosta et al., 2007; Ciccarelli et al., 2009), and with clinical (Ellis et al., 1999; Abe et al., 2004; Filippini et al., 2010) and electrophysiological (Sach et al., 2004; Iwata et al., 2008) measures of UMN degeneration. Conversely, the disease duration was negatively correlated with FA in tracts outside the CST (Thivard et al., 2007; Keil et al., 2012), suggesting again that the impairment of motor structures is an early phenomenon, which is followed by the impairment of extra-motor networks.

DTI has been also used to examine differences across clinical phenotypes. Patients with bulbar-onset ALS have the most marked cerebral FA decreases (Ellis et al., 1999; Aoki et al., 2005; van der Graaff et al., 2011). Two voxel-based DTI studies of patients with PMA showed decreased FA in the white matter underneath the right primary motor cortex (van der Graaff et al., 2011) and the corticospinal tract (Sach et al., 2004), including those patients that later developed ALS (Sach et al., 2004), suggesting that DTI might also be a marker for early and clinically silent UMN involvement. However, a more recent study failed to find differences in DTI metrics in PMA patients, although in those with cognitive impairment, changes in extra-motor tracts could be detected (Spinelli et al., 2016). One study compared ALS and PLS patients and found that PLS patients had lower FA in the body of the corpus callosum and in the white matter adjacent to the right precentral motor cortex, while ALS patients had reduced values adjacent to the superior frontal gyrus (Ciccarelli et al., 2009).

Only few longitudinal studies have focused on DTI changes over time. Most studies have suggested that DTI metrics are sensitive to disease progression (Jacob et al., 2003; Nickerson et al., 2009; Agosta et al., 2010b; van der Graaff et al., 2011; Zhang et al., 2011; Keil et al., 2012; Menke et al., 2012). FA was found to decrease in a progressive and linear manner along the CST (Jacob et al., 2003; Sage et al., 2007; Nickerson et al., 2009) while the diffusivity of the external and internal capsule was increased (Keil et al., 2012). To be considered, one longitudinal study showed that white matter defects progressed slower than those at the basal ganglia (Menke et al., 2014).

*In summary*, the most promising DTI biomarker candidate for diagnosis and follow-up evaluation is the decreased FA in the corticospinal tract and corpus callosum. Furthermore, DTI may allow ALS phenotyping by detecting white matter tract changes in motor and extra-motor regions. Tract-based spatial statistics method for analysis of DTI metrics is reccomended, and FA and mean diffusivity of the CST and corpus callosum should be included within the structured report.

### Magnetic Resonance Spectroscopy

Magnetic Resonance Spectroscopy (MRS) is an analytical method that enables the identification and quantification of regional brain metabolites. Many nuclei may be used to obtain their MR spectra. Proton (^1^H-MRS) is widely used for clinical MRS because of its natural abundance in living tissue and its high nuclear MR sensitivity. The MR spectrum is represented by a graph in which the x-axis denotes the different chemical frequencies of the full sample in parts per million while the y-axis relates to their concentrations. Spectra are obtained either from one selected brain region, in the case of single-voxel spectroscopy, or from multiple voxels in the case of MR spectroscopic imaging. Three-dimensional echo-planar spectroscopic imaging allows the volumetric analysis by using echo-planar readouts to accelerate spatial encoding (Ebel et al., 2001). Although more than 30 metabolites can be distinguished, the most commonly investigated are: N-acetyl aspartate (NAA), a marker of neuronal integrity; choline (Cho), a marker of cell membrane turnover; and creatine (Cr), a marker of energy metabolism. Other reported metabolites include: myo-inositol (mI), a marker of glial cells; glutamate and glutamine (collectively termed Glx), a marker of the glutamatergic neurotransmitter system; and γ-aminobutyric acid (GABA), the major inhibitory neurotransmitter. Metabolite quantification can be expressed in the form of ratios or absolute concentrations. Ratios are frequently used because normalization of the metabolites relative to either Cr or Cho corrects for variability, including magnetic field inhomogeneity and volume loss. Although absolute quantification is generally considered to be more informative, it requires additional measurements.

MRS studies in ALS have consistently demonstrated a decrease in absolute NAA concentrations (Gredal et al., 1997; Rooney et al., 1998; Bradley et al., 1999; Pohl et al., 2001b; Sarchielli et al., 2001; Schuff et al., 2001; Block et al., 2002; Suhy et al., 2002; Kalra et al., 2006a,c; Mitsumoto et al., 2007; Han and Ma, 2010; Cervo et al., 2015; Liu et al., 2015) or NAA/Cr (Pioro et al., 1994; Jones et al., 1995; Block et al., 1998, 2002; Rooney et al., 1998; Abe et al., 2001; Rule et al., 2004; Yin et al., 2004; Kalra et al., 2006a,b; Mitsumoto et al., 2007; Charil et al., 2009; Lombardo et al., 2009; Han and Ma, 2010; Pyra et al., 2010; Sivák et al., 2010), NAA/Cho (Pioro et al., 1994; Jones et al., 1995; Giroud et al., 1996; Block et al., 1998, 2002; Rooney et al., 1998; Pohl et al., 2001a; Suhy et al., 2002; Kalra et al., 2006a; Rule et al., 2004; Pyra et al., 2010), NAA/(Cr + Cho) (Rooney et al., 1998; Suhy et al., 2002; Rule et al., 2004) ratios in the motor cortex, most probably related to neurodegeneration. Additionally, other investigated motor cortex metabolites have shown an increased in Glu/Cr and Glx/Cr ratios (Han and Ma, 2010), increased mI (Bowen et al., 2000) or mI/Cr ratio (Block et al., 1998; Kalra et al., 2006a; Lombardo et al., 2009), and decreased GABA (Foerster et al., 2012a, 2014). The reduced GABA levels suggest that GABAergic dysfunction could have an important role in ALS neurodegeneration. Additionally, low levels of NAA and decreased NAA ratios have been observed in the corona radiata and periventricular white matter (Yin et al., 2004; Pyra et al., 2010), in the posterior limb of internal capsule (Schuff et al., 2001; Han and Ma, 2010) and in the entire CST tract (Rooney et al., 1998; Pyra et al., 2010; Govind et al., 2012; Foerster et al., 2014). MRS studies have also found decreased NAA ratios in the thalamus and basal ganglia (Sharma et al., 2011), mid-cingulate cortex (Sudharshan et al., 2011), frontal, parietal, and occipital lobes (Bowen et al., 2000; Abe et al., 2001; Rule et al., 2004; Sivák et al., 2010).

The methodological variability and the considerable overlap between patients and controls limit the clinical value of MRS in daily practice. The combination of MRS with transcranial magnetic stimulation or DTI showed potential to improve the sensitivity to detect UMN degeneration (Pohl et al., 2001b; Kaufmann et al., 2004). Several studies have reported moderate correlations between several metabolite concentration changes and clinical measures of UMN deficits (Rooney et al., 1998; Bowen et al., 2000; Block et al., 2002; Wang et al., 2006b; Mitsumoto et al., 2007), disease severity (Ellis et al., 1998; Abe et al., 2001; Wang et al., 2006b), lateralization of clinical symptoms (Block et al., 2002), disease duration and progression (Pohl et al., 2001a; Pyra et al., 2010). Also, a decreased frontal NAA/Cr ratio has been associated with executive function deficits (Abe et al., 2001; Quinn et al., 2012).

Interestingly, lower NAA/Cr ratios have been reported across different motor neuron disease phenotypes (ALS, PLS, and PMA), albeit changes in PMA patients seem to be milder (Mitsumoto et al., 2007). Bulbar onset patients have lower motor cortex NAA and NAA/Cr ratio than spinal onset patients (Ellis et al., 1998). Patients with severe bulbar weakness or spasticity have low NAA/Cr ratio in the brainstem, while it is almost normal in patients with predominantly LMN weakness (Unrath et al., 2007). Furthermore, a low NAA/Cho ratio has been associated with reduced survival (Kalra et al., 2006c).

Longitudinal MRS studies have shown decrease in the NAA peak and NAA/Cr and NAA/Cho ratios in the primary motor cortex over follow-up periods of up to 28 months (Block et al., 1998, 2002; Pohl et al., 2001a; Suhy et al., 2002; Rule et al., 2004; Unrath et al., 2007). Changes in NAA concentrations are probably associated with disease progression (Pohl et al., 2001a; Unrath et al., 2007). Furthermore, while NAA, Cr, and Cho concentrations decreased over time in the motor cortex, concentrations in non-motor regions remained qualitatively unchanged. Finally, MRS has been used to monitor the therapeutic effects of riluzole, creatine, and minocycline in small cohorts of patients with ALS (Kalra et al., 1998, 2006b; Vielhaber et al., 2001; Atassi et al., 2010; Khiat et al., 2010) although the absence of an untreated patient arm in most of them limits the interpretation of their findings.

*As a summary*, MRS may have a potential role in the diagnosis of ALS when combined with other MR biomarkers. Although there is no consensus on which metabolites or ratios accurately reflect UMN involvement, the most promising one is motor cortex NAA. Glx, mI, and GABA candidates for diagnostic biomarkers require additional research. Several studies have reported correlations between metabolite concentration changes and clinical measures of UMN deficits, severity of disease, disease duration, disease progression, and response to treatment. The development of comparatively high-spatial resolution whole-brain MRS imaging and automated processing software might offer renewed potential as part of a multimodal biomarker panel. Moreover, ultra-high-field MRS (7 Tesla) will improve the sensitivity and spectral resolution (Donatelli et al., 2018a), allowing GABA measurements further exploring ALS pathogenesis.

### Quantitative Iron Imaging

Brain iron can be quantified using relaxometry. The three primary relaxometry metrics pertinent to the detection of brain iron are the transverse relaxation rates or reciprocals of the transverse relaxation times (R2 = 1/T2, R2^∗^ = 1/T2^∗^, and R2′ = 1/T2′). Moreover, an automated procedure that allows analyzing R2 relaxation rates on a voxel-by-voxel basis, known as voxel-based relaxometry (VBR), has been developed. It has been demonstrated that the R2^∗^values are correlated to the amount of iron determined chemically on postmortem specimens (Langkammer et al., 2010b). Few studies have analyzed the iron content in the brain of ALS patients using relaxometry methods. One study found increased R2^∗^ in the caudate nucleus and a trend for R2^∗^ values to be elevated in the CST of ALS patients (Langkammer et al., 2010a). Moreover, a reduced relaxation rate in the right precentral gyrus and ventral pons, corresponding to the corticospinal tract, was observed in a VBR study (Minnerop et al., 2009).

Although SWI does not directly provide quantitative measures of magnetic susceptibility, the SWI phase shifts can be used to quantitatively measure iron content in the brain (Haacke et al., 2007). A recent developed technique is the quantitative susceptibility mapping (QSM), while SWI generates contrast based on phase images, QSM further computes the underlying susceptibility of each voxel as a scalar quantity. A retrospective study of iron deposition in motor cortex found that QSM was more accurate than T2^∗^, T2 or FLAIR sequences (Schweitzer et al., 2015). Moreover, increased magnetic susceptibility in the motor cortex has been correlated with UMN impairment (Costagli et al., 2016). Another QSM study also found changes in the corpus callosum, along the corticospinal tract and in the subgyral regions of frontal, parietal, temporal, occipital and limbic lobes, being the alterations in the corpus callosum correlated with disease severity (Prell et al., 2015). Finally, ultra-high-field imaging has a better sensitivity to susceptibility; it is superior to quantify the magnetic susceptibility of tissues with QSM, and their results are expected to be more accurate (Donatelli et al., 2018a).

**FIGURE 5:**
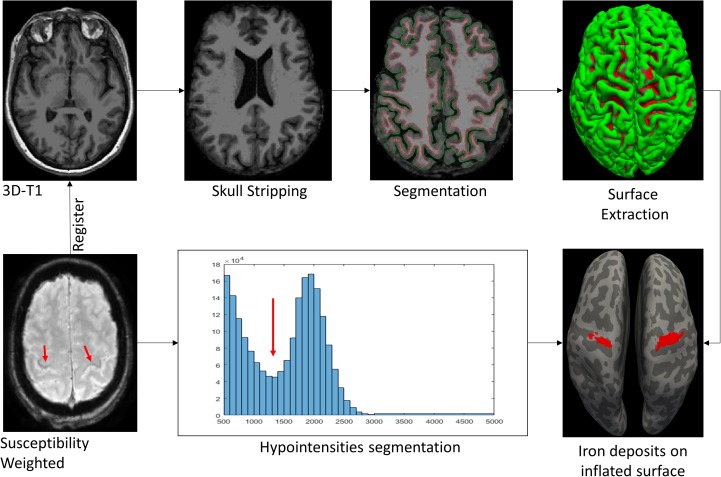
MR pipeline followed in our center to highlight the iron deposits. Susceptibility Weighted (SW) images are registered to the T1 space. Then, skull stripping is obtained from 3D T1-weighted images. The segmentation process, based on atlas and threshold techniques, isolate the white matter (WM) and gray matter (GM). Surface extraction obtains the frontiers between WM-GM (red line) and WM-pia (green line). The 3D inflated pial surface reconstruction improves the sulci (light gray) and gyrus (dark gray) visualization. Finally, iron deposits from SW images are matched over the inflated surface.

*In conclusion*, quantitative iron imaging is a very promising biomarker in ALS. Several studies have reported correlations between iron overload concentration and clinical measures of UMN deficits. Iron cortical maps are recommended to be included within the structured report (Figure 5).

### Functional Magnetic Resonance (fMR) Imaging

Brain function can be studied *in vivo* by fMR imaging based on the blood oxygenation level-dependent (BOLD) contrast. BOLD imaging relies on regional differences in the cerebral blood flow and desoxyhemoglobine content to delineate regional brain activity. The technique uses a gradient-echo sequence sensitive to the oxygenation level in blood, which reflects the imbalance between oxy- and deoxyhemoglobin concentrations. fMR signal detect subtle alteration in response to stimuli or actions (task) or when the patient is not performing an explicit task (resting state, rs). Task-based fMR imaging examines one domain and its related network, while rs-fMR imaging simultaneously searches all networks and inter-relationships. In the rs-fMR exams, functional connectivity expresses the intrinsic random fluctuations related to spontaneous brain activity. rs-fMRI data is usually analized by seed-based and independent component analysis (ICA). During seed-based analysis, data is circumscribed to the previously defined voxles while the ICA data-driven approach separates signals into non-overlapping spatial components according to their time courses. The ICA methods have the advantage of being fully automated and observer-independent (Agosta et al., 2010a). With rs-fMR exams, 10-to-20 consistent networks have been described, including the default mode network (Barkhof et al., 2014).

#### Task-Based fMRI

During motor tasks, fMRI has demonstrated heightened activation of primary motor cortex (Kollewe et al., 2011; Mohammadi et al., 2011, 2015), premotor and supplementary motor areas (Konrad et al., 2002; Han and Ma, 2006; Lulé et al., 2007b; Stanton et al., 2007; Cosottini et al., 2012), sensorimotor cortex (Konrad et al., 2002; Han and Ma, 2006; Stanton et al., 2007; Mohammadi et al., 2011), temporo-parietal associative sensory areas (Konrad et al., 2002, 2006; Schoenfeld et al., 2005; Stanton et al., 2007; Cosottini et al., 2012), and cerebral regions involved in motor learning (basal ganglia and cerebellum) (Schoenfeld et al., 2005; Konrad et al., 2006). These results complement previous results noted using PET (Kew et al., 1993). Such functional reorganization might represent cortical plasticity in response to loss of pyramidal cells in the primary motor cortex (Schoenfeld et al., 2005) or reduced local inhibitory interneuronal function (Kew et al., 1993; Turner and Kiernan, 2012).

Some studies have suggested a relation between the increase in movement-associated cortical activations beyond the primary motor cortex and the degree of UMN involvement (Tessitore et al., 2006; Mohammadi et al., 2011). Moreover, activation of extra-motor areas during motor tasks is lower in patients with faster disease progression over 1 year than in patients with slower disease progression (Poujois et al., 2013).

Bulbar onset patients have shown low activation of the motor cortex compared with limb onset patients, suggesting two different patterns of cortical activation changes and a lack of compensatory capacity for bulbar movements (Li et al., 2009; Mohammadi et al., 2009b; Kollewe et al., 2011).

Cerebral activation alterations have also been described when undertaking visual tasks (Lulé et al., 2010), executive and language tasks (Abrahams et al., 2004; Lulé et al., 2010) and processing of socioemotional stimuli (Lulé et al., 2007a; Palmieri et al., 2010; Passamonti et al., 2013). Overall, fMRI data present functional evidence for multisystem involvement of cognitive, emotional and sensory processing pathways in ALS (Grolez et al., 2016).

#### Resting State fMRI

In ALS patients, the rs-fMR studies have demonstrated changes in the premotor cortex and a widespread reorganization of the functional connectivity. Searching for differences between patients with ALS and controls, most rs-fMR studies have revealed decreased functional connectivity within the sensorimotor network (Mohammadi et al., 2009a; Jelsone-Swain et al., 2010; Tedeschi et al., 2012; Fekete et al., 2013; Zhou et al., 2013) and abnormalities in other networks related to cognition and behavior, including the default mode network (Mohammadi et al., 2009a; Jelsone-Swain et al., 2010; Luo et al., 2012; Zhou et al., 2013). However, other studies have shown an increased network coherence in the somatosensory and extra-motor areas (Agosta et al., 2011, 2013, 2014; Douaud et al., 2011; Luo et al., 2012; Tedeschi et al., 2012; Fekete et al., 2013; Zhou et al., 2013). Interestingly, this pattern of increased sensorimotor networks functional connectivity is more widespread in patients with preserved DTI in the CST than patients with severe CST damage (Agosta et al., 2011). Two hypotheses can explain these divergent results with disease progression. On the one hand, that this increased functional connectivity represents the compensation for structural damage (Agosta et al., 2011), being exhausted when the burden of pathology increases. On the other hand, that it represents the reduced local inhibitory function (Turner and Kiernan, 2012), which characterizes the early disease, as supported by the MRS GABA concentrations decrease in the primary motor cortex (Foerster et al., 2013a) and decreased uptake of ^11^C-flumazenil in the primary motor cortex and frontal cortex by means of PET (Zhu et al., 2015).

Additionally, the association between DTI structural connectivity and rs-fMR functional connectivity changes indicates that both structural and functional network degeneration in ALS are coupled (Schmidt et al., 2014). Interestingly, both the structural connectome emerging from motor regions and the functional network expansion, resemble the pathological staging of TDP-43 (Schulthess et al., 2016). Simulating disease propagation across white matter connectome reveals anatomical substrate for neuropathology staging in ALS. However, a sequential spreading at the individual level could not be shown.

Finally, the connectivity between the left sensorimotor cortex, right parahippocampal gyri and fourth cerebellar lobule have been correlated with disease severity (Agosta et al., 2011). The activity in the right parahippocampal gyri and connectivity in the frontal cortex have been correlated with disease progression and duration (Luo et al., 2012; Zhu et al., 2015), while activity in the left anterior cingulate and left temporal gyrus has been correlated with cognitive parameters (Zhu et al., 2015). The changes in the hippocampal region, including its connectivity, are related to its dysfunction; and the dysfunction contributes to the clinical heterogeneity of ALS (Christidi et al., 2018).

*In summary*, while task-based fMR imaging has important limitations in ALS patients, because their limited ability to perform a given task, rs-fMR offers the advantages of spontaneous and simultaneously analysis of brain activity within all networks and their inter-relationships. Moreover, rs-fMR exams might provide valuable information on ALS pathogenesis (functional reorganization and disease spreading) and confirms that ALS is a multisystem disease not limited to motor areas.

## Applications of the MR Imaging Biomarkers in ALS

MR imaging biomarkers have already improved our understanding of pathophysiology and the mechanisms underlying the progressive degenerative process in ALS. However, the most important remaining challenge is their integration into clinical trials and practice, as surrogate markers for the diagnosis, stratification, and monitoring of the disease progression. Up to now, although promising, results are very variable and no firm conclusions can be drawn. The small samples, suboptimal patient characterization, and lack of standardization of the schemes and analysis procedures, are the main challenges to implement MR imaging biomarkers in the clinical practice (Menke et al., 2017).

### Role as Diagnostic Biomarkers

Qualitative biomarkers have been traditionally found unsensitive and unspecific for the ALS diagnosis (Agosta et al., 2010a). However, the reliable semi-quantitative assessment of iron-related hypointensities in the motor cortex may improve the sensitivity of the research diagnostic criteria and could help to the diagnosis in some ALS phenotypes in the clinical practice.

Regarding quantitative biomarkers, most studies have focused on group results using healthy subjects as controls, limiting its translation into clinical practice. Up to now, only one study has assessed the diagnostic accuracy of multimodal structural MR using ALS mimics, finding a sensitivity of 75% and a specificity of 92%, when both cortical thickness and callosal DTI measures were (Ferraro et al., 2017). The combination of cortical thickness and iron-related signal intensity could also be used to increase the diagnostic accuracy of each independent measure (Donatelli et al., 2018b).

### Role as Stratification Biomarkers

At present, MR imaging biomarkers are not superior to the clinical evaluation for patient stratification (Menke et al., 2017). However, a recent MR study identified more extensive brain structural changes in rapid vs. slow progression patients, suggesting that a MR based phenotyping could, in the future, also offer prognostic information (Senda et al., 2017).

### Role as Progression Biomarkers

Neuroimaging biomarkers offer several advantages over clinical milestones, when evaluating the outcomes of a clinical trial (Menke et al., 2017). They are more objective, there is no placebo effect, and they measure exclusively a disease-modifying effect (not a symptomatic effect). However, the lack of standardization in the longitudinal studies and between centers has resulted in very heterogeneous results, difficulting their implementation in multicenter studies (Menke et al., 2017). Moreover, they have been shown less powered that clinical measures to detect changes in ALS patients, and consequently MRI biomarkers have not been incorpored into clinical trials up to now. Interestingly, subcortical and extramotor structures are the most frequent longitudinal changes (Menke et al., 2014, 2017; Westeneng et al., 2015; Cardenas-Blanco et al., 2016), suggesting that these regions could be more appropriate as progression biomarkers.

## Conclusion

Brain MR imaging is a widely available non-invasive technique, able to provide both structural and functional information.

MR biomarkers have already greatly contributed to disentangle ALS pathophysiology. Multimodality analysis, combining various advanced neuroimaging techniques, may be an interesting option for implementing biomarkers into the clinical practice and clinical trials. To that end, the most promising metrics to assess the motor system damage seem to be cortical thickness and iron overload quantification in the motor cortex, and FA of the cortical spinal tract and corpus callosum.

Contextual structured reporting (disease-specific) has many advantages including increased clarity, specific, complete content, and increased ability to mine data for research. In the ALS-structured report, we suggest to include at least a visual assessment of the motor cortex and CST on SWI and FLAIR respectively, cortical thickness of the motor cortex, and FA and radial diffusivity of the CST and corpus callosum. These metrics have already been used in scientific research and they are increasingly being applied to diagnose and phenotype patients in clinical practice (Ferraro et al., 2017). Adding a semi-quantitative assessment of the iron content in the motor cortex would probably increase the sensitivity and specificity to the multimodal analysis, although this deserves further investigation. Network-based system analyses could integrate information about these biomarkers in both ALS patients and controls into machine learning algorithms. Thus, in the future, these algorithms might inform about diagnosis, prognosis or progression at an individual level. Last but not least, regarding technological advances, the introduction of ultra-high field MR imaging will improve the resolution and accuracy of the already implemented techniques.

## Author Contributions

All authors defined, wrote, reviewed, and approved the manuscript. MM, JVC and LM-B also performed the clinical research in ALS patients.

## Conflict of Interest Statement

The authors declare that the research was conducted in the absence of any commercial or financial relationships that could be construed as a potential conflict of interest.
